# Double-wire woven nitinol stent for treating dogs with refractory tracheal collapse: A case series

**DOI:** 10.17221/61/2023-VETMED

**Published:** 2024-01-23

**Authors:** Jin-Young Choi, Mu-Young Kim, Hun-Young Yoon

**Affiliations:** ^1^Department of Veterinary Surgery, College of Veterinary Medicine, Konkuk University, Gwangjin-gu, Seoul, Republic of Korea; ^2^Department of Veterinary Clinical Sciences, College of Veterinary Medicine, Purdue University, West Lafayette, IN, USA; ^3^KU Center for Animal Blood Medical Science, Konkuk University, Gwangjin-gu, Seoul, Republic of Korea

**Keywords:** canine tracheal stent, self-expanding nitinol stent, stent fracture

## Abstract

This retrospective case series describes the signalments, clinical signs, diagnostic test results, and postoperative complications obtained from the medical records of 10 client-owned dogs that underwent treatment for grade IV tracheal collapse using double-wire woven nitinol stents between October 2017 and September 2021. Respiratory signs resolved in all dogs immediately after tracheal stent placement. Mild to moderate stent fractures were identified in five dogs, of which two showed concurrent respiratory distress necessitating re-stenting after several months. Minor complications, such as stent migration, were absent. The double-wire woven nitinol stent optimised for the canine trachea showed favourable outcomes and minimal complications.

Tracheal collapse is a progressive disorder in which the tracheal cartilage weakens and the tracheal lumen cannot be maintained and collapses, resulting in death (Della Maggiore 2020). Dogs with end-stage tracheal collapse develop clinical signs, such as a goose-honking cough, open-mouth breathing, paradoxical abdominal movement, cyanosis, and dyspnoea, which may be fatal if they lead to respiratory distress (Durant et al. 2012; MacPhail 2018). Recently, intraluminal stents have been widely used instead of surgical methods, including extraluminal stent placement, because of the simple procedure, shorter anaesthesia time, and immediate recovery and discharge after the procedure (Durant et al. 2012).

While intraluminal stent placement has several advantages, complications have been documented, including stent fracture (19–50%) or migration (8–37%), excessive granulation tissue formation, aspiration pneumonia, and tracheitis (21–58%) (Sura and Krahwinkel 2008; Durant et al. 2012; Weisse et al. 2019). To minimise such complications, the selection of an appropriately sized stent with suitable mechanical properties, the use of high-quality surgical techniques, and the treatment of underlying diseases are essential.

Based on the mechanical properties and clinical results from previous studies on self-expanding metal stents (Kim et al. 2022a; Kim et al. 2023), it was determined that a D-type stent with a wire diameter of 0.006 (D6) would be most suitable for the treatment of canine tracheal collapse.

This case series illustrates some of the major complications and clinical outcomes obtained through long-term follow-up after the application of canine-optimised stents in dogs diagnosed with end-stage, refractory grade IV tracheal collapse.

## Case description

All included dogs were admitted to a veterinary teaching hospital between October 2017 and September 2021. The research was approved by the Institutional Animal Care and Use Committee. The signalment and diagnosis-related information of the 10 dogs obtained before surgery are presented in Table 1. The stent size, complications, number of stent placements, survival time, and cause of death are listed in Table 2.

**Table 1:** Signalment, diagnosis, and underlying disease of the 10 dogs treated with tracheal stents

Dog	Breed	Age (years)	Sex	b.w. (kg)	TC clinical grade	Fluoroscopic findings	Underlying disease
1	Yorkshire Terrier	14	IF	3	TC dynamic G4	BC	MMVD D, pulmonary oedema, pneumonia, CKD III, pancreatitis
2	Pomeranian	8	CM	3.3	TC static G4	lung herniation	soft palate elongation, thickening
3	Miniature Pinscher	15	CM	2.5	TC static G4 (TI)/G3 (IT)	BC	*pectus excavatum*
4	Pomeranian	16	SF	6.7	TC dynamic G4	BC	MMVD C, enteritis, chronic pancreatitis
5	Chihuahua	8	CM	2	TC dynamic G4	lung herniation	*pectus carinatum*
6	Yorkshire Terrier	9	SF	2	TC static G4 (TI)	BC, lung herniation, tracheal kinking	soft palate elongation, nasopharyngeal collapse
7	Pomeranian	16	CM	3.75	TC dynamic G4	BC, lung herniation, tracheal kinking	CKD III, laryngeal saccule
8	Poodle	7	SF	3.3	TC static G4	lung herniation, tracheal kinking	*pectus excavatum*
9	Pomeranian	8	CM	3.1	TC dynamic G4	BC, lung herniation, tracheal kinking	MMVD C, soft palate elongation, thickening
10	Pomeranian	7	CM	3.5	TC dynamic G4	BC, lung herniation, tracheal kinking	*pectus carinatum*

b.w = body weight; BC = bronchial collapse; CKD = chronic kidney disease; CM = castrated male; G4 = grade 4; IF = intact female; MMVD = myxomatous mitral valve degeneration; SF = spayed female; TC = tracheal collapse

**Table 2:** Ten dogs grouped into mild and severe types according to the presence of tracheal kinking

Dog	Stent size (mm)	Complication (Op)	Fracture type	No. of stents	Weight change (%)	Survival time (days)	Cause of death
1	12 × 80	–	0	1	0	82	congestive heart failure
2	12 × 80	–	0	1	0	1 730	(alive)
3	14 × 70	–	0	1	0	480	cardiac arrest
4	12 × 80	–	0	1	–0.2 kg	874	(alive)
5	8 × 60	–	0	1	–0.3 kg	955	(alive)
6	8 × 70	stent fx (723)	2	1	1 kg (50)	753	respiratory distress
7	14 × 80	stent fx (340)	2	1	0.5 kg (13)	705	pancreatitis
8	12 × 70	collapse (330/209/80)	1	4	0.7 kg (21)	659	respiratory distress
9	10 × 80	stent fx (263)	2	1	0.1 kg (3)	430	congestive heart failure
10	10 × 80	stent fx (533)	2	2	1 kg (28)	741	(alive)

fx = fracture; Op = postoperative days

Data on the size of the stent used, complications of the stent, weight change after stent placement, and survival time

Stent placement was indicated for dogs with no improvement in clinical signs after more than 4 weeks of medical treatment for tracheal collapse. Tracheal and principal bronchial movements during respiration were dynamically evaluated and the grade of tracheal collapse was assessed using fluoroscopy. Stent size was selected using static thoracic radiography under the animal’s natural respiration. The distance from 1 cm posterior to the cricoid cartilage, to 1 cm anterior to the carina was defined as the stent length. A total of 110–120% of the maximum diameter of the corresponding trachea was selected as the stent diameter.

Butorphanol (0.1 mg/kg, i.v.; Butorphan^®^; Myungmoon Pharmaceutical Co., Seoul, Republic of Korea) was administered as a premedication. Propofol (6 mg/kg, i.v., Provive^®^ 1%; Myungmoon Pharmaceutical Co., Seoul, Republic of Korea) was used to induce anaesthesia. After intubation, general anaesthesia was maintained with isoflurane (Isoflurane^®^; Choongwae Co., Seoul, Republic of Korea) and oxygen.

An intraluminal double-wire woven nitinol stent [Niti-S Biliary Uncovered Stent (D-type); Taewoong Medical] was manufactured and used in the study. Stent placement was performed using methods described in previous studies (Yoon et al. 2017; Kim et al. 2022b). The average stenting duration in all dogs was 12 min (range, 5–15 min), and the average anaesthesia time was 25 min (range, 10–40 min). Immediately after stent placement, signs of dyspnoea and goose-honk coughing resolved in all dogs.

For postoperative sedation and analgesia, butorphanol (0.1–0.2 mg/kg, i.v., every 12 h) was administered. Prednisolone (0.5 mg/kg, p.o., every 2–4 h); Solondo Tap (Yuhan Co., Chungbuk, Republic of Korea) was also administered for one week. Amoxicillin with clavulanate (11 mg/kg, p.o., every 12 h, Amocla Tap; Kuhnil Pharmaceutical Co., Jung-gu, Republic of Korea) was prescribed for 7–14 days to prevent secondary infections. The length of hospital stay was 1–3 days for all dogs.

Reassessments were conducted after discharge, and the degree of fracture was radiographically evaluated using the stent fracture grading system as follows (Allie et al. 2004; Scheinert et al. 2005): 0 = normal, no stent fracture; 1 = mild fracture, single strut fracture only; 2 = moderate fracture, multiple single-stent fractures; 3 = severe fracture, complete separation of the stent segment (Scheinert et al. 2005).

**Figure 1:**
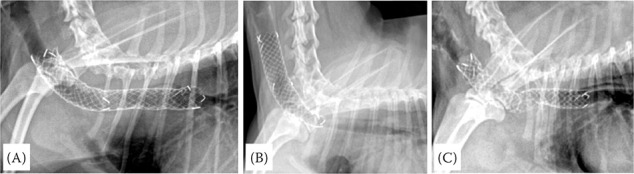
Lateral thoracic radiograph view showing types of stent fracture during inspiration (A) Mild stent fraying shown on day 251 in Dog 3 (yellow arrow). (B) Type 1 stent fracture identified on day 220 in Dog 8. A segmental stent was placed from the cervical trachea to the thoracic inlet. (C) Type 2 stent fracture identified on day 533 in Dog 10

Dogs 3 and 8 experienced stent fraying, an indication of a minor stent strut fracture, but this was not associated with clinical signs (Sura and Durant 2012) (Figure 1A). Five dogs with tracheal kinking showed mild to moderate stent fracture and a 3–50% increase in body weight.

Re-stenting was performed in two dogs, on day 330 after initial stenting in Dog 8 with a grade 1 fracture (Figure 1B) and on day 533 in Dog 10 with a grade 2 fracture (Figure 1C). A total of four stents were placed in Dog 8; the re-stenting periods were 330, 209, and 80 days and the survival period was 659 days.

Two dogs, Dogs 6 and 8, died from respiratory distress. The causes of death for the other dogs were congestive heart failure, pancreatitis, and cardiac arrest due to worsening underlying diseases.

## DISCUSSION

This case series report describes the outcome of canine-optimised tracheal stent placement in dogs with grade IV tracheal collapse refractory to medical therapy.

In dogs with severe tracheal collapse, a stent is used to support the tracheal wall against excessive mechanical stress, as the cartilage is weakened and can no longer support the tracheal wall as in healthy dogs. The more severe the cough and chronic tracheitis, the more clinical signs and damage to the tracheal wall and mucosa, leading to a reduced ability of the tracheal cartilage to support the lumen. In particular, intense pressure in the thoracic cavity occurs while coughing. In dogs with end-stage tracheal collapse, in which the physical properties of the trachea are poor, the trachea loses its straightened shape because it cannot withstand the pressure acting on the lumen during coughing, resulting in a phenomenon called tracheal kinking in severe cases. In this study, five of the 10 dogs showed tracheal kinking on fluoroscopy, and the stent fracture rate was high.

Being overweight in dogs with tracheal collapse significantly increases the risk of relapsing respiratory signs. In the present study, Dog 10 showed a 50% body weight increase after the first procedure and the highest grade of stent fracture (grade 2). As weight increases, adipose tissue accumulates in the dorsal muscles surrounding the trachea, causing tracheal and bronchial collapse to worsen (de Oliveira Lemos et al. 2020; Song et al. 2022). Five dogs in this study had a postoperative weight gain of 3–50% and showed mild to moderate stent fractures. Weight gain worsens the pathological conditions in dogs with tracheal collapse and influences the tracheal stents. Theoretically, when the tracheal radial force is increased, stent fatigue is increased by placing more significant pressure on the stent supporting the lumen of the trachea. This can eventually increase the possibility of fractures; thus, weight control is crucial to reducing stent fractures in all stented dogs.

Stent fracture is defined as the complete or partial separation of the embedded stent that maintains the luminal patency of the organ. In human medicine, several studies have classified stent fractures for vascular stents and evaluated the degree of stent occlusion, restenosis, and fracture, and found that there is no correlation with mortality (Scheinert et al. 2005; Kan et al. 2016). In veterinary medicine, there is no indication for predicting factors related to clinical signs or mortality rate according to stent fracture type to date. In the present study, the degree of fractures of canine tracheal stents was compared, referring to the morphological changes of stents in human medicine. Recurrence of respiratory signs to the extent requiring re-stenting was confirmed in dogs with mild or moderate stent fractures. Further studies related to prognosis are needed on methods to evaluate stent fracture grade in patients with canine tracheal stents.

In summary, a canine-optimised tracheal stent was applied in dogs with grade IV tracheal collapse in this study, with a low major complication rate and favourable clinical outcomes. Dogs with tracheal kinking confirmed by fluoroscopy and those with weight gain after stent placement were vulnerable to stent fracture and thus had a poor prognosis. A grading system for stent fractures could be helpful for evaluating the changes in morphologic details in a follow-up procedure.

## References

[R1] Allie DE, Hebert CJ, Walker CM. Nitinol stent fractures in the SFA. Endovasc Today. 2004 Jul/Aug:22-34.

[R2] Della Maggiore A. An update on tracheal and airway collapse in dogs. Vet Clin North Am Small Anim Pract. 2020 Mar;50(2):419-30. 31864678 10.1016/j.cvsm.2019.11.003

[R3] de Oliveira Lemos NM, Santos Filho M, Mendes CD, do Carmo JS, Eleuterio EO, da Veiga CC, Paiva JP. Influence of obesity on the clinical improvement of tracheal and bronchial collapse in dogs: A case report. Braz J Vet Res Anim Sci. 2020;42(1):e107620.

[R4] Durant AM, Sura P, Rohrbach B, Bohling MW. Use of nitinol stents for end-stage tracheal collapse in dogs. Vet Surg. 2012 Oct;41(7):807-17. 22957667 10.1111/j.1532-950X.2012.01037.x

[R5] Kan J, Ge Z, Zhang JJ, Liu ZZ, Tian NL, Ye F, Li SJ, Qian XS, Yang S, Chen MX, Rab T, Chen SL. Incidence and clinical outcomes of stent fractures on the basis of 6 555 patients and 16 482 drug-eluting stents from 4 centers. JACC Cardiovasc Interv. 2016 Jun 13;9(11):1115-23. 27009464 10.1016/j.jcin.2016.02.025

[R6] Kim JH, Choi JY, Yoon HY. Evaluation of mechanical properties of self-expanding metal stents for optimization of tracheal collapse in dogs. Can J Vet Res. 2022a Jul;86(3):188-93. 35794973 PMC9251794

[R7] Kim JH, Choi JY, Yoon HY. Comparison of three different self-expanding metal stents using rabbit models for the treatment of tracheal collapse. Acta Cir Bras. 2022b Aug 12;37(5):e370502. 35976340 10.1590/acb370502 PMC9377205

[R8] Kim JH, Choi JY, Yoon HY. A rabbit model of tracheal collapse for optimal self-expanding metal stents. J Vet Med Sci. 2023 Mar 28;85(3):386-92. 36740259 10.1292/jvms.22-0167 PMC10076205

[R9] MacPhail C. Surgery of the upper respiratory system. In: Fossum TW, editor. Small animal surgery. 5^th^ ed. Philadelphia, PA: Mosby; 2018. p. 833–3.

[R10] Scheinert D, Scheinert S, Sax J, Piorkowski C, Braunlich S, Ulrich M, Biamino G, Schmidt A. Prevalence and clinical impact of stent fractures after femoropopliteal stenting. J Am Coll Cardiol. 2005 Jan 18;45(2):312-5. 15653033 10.1016/j.jacc.2004.11.026

[R11] Song Y, Kim Y, Kim J, Kim KN, Oh S, Kim HJ. Management of pulmonary hypertension due to brachycephalic obstructive airway syndrome in a dog. J Vet Clin. 2022 Oct 31;39(5):240-5.

[R12] Sura P, Durant AM. Trachea and bronchi. In: Tobias KM, Johnston SA, editors. Veterinary surgery: Small animal. 1^st^ ed. Vol 2. St. Louis, Missouri: Elsevier Saunders; 2012. p. 1746-50.

[R13] Sura PA, Krahwinkel DJ. Self-expanding nitinol stents for the treatment of tracheal collapse in dogs: 12 cases (2001–2004). J Am Vet Med Assoc. 2008 Jan 15;232(2):228-36. 18275390 10.2460/javma.232.2.228

[R14] Weisse C, Berent A, Violette N, McDougall R, Lamb K. Short-, intermediate-, and long-term results for endoluminal stent placement in dogs with tracheal collapse. J Am Vet Med Assoc. 2019 Feb 1;254(3):380-92. 30668235 10.2460/javma.254.3.380

[R15] Yoon HY, Choi JW, Ji HK, Jung HK. Use of a double-wire woven uncovered nitinol stent for the treatment of refractory tracheal collapse in a dog: A case report. Vet Med-Czech. 2017 Feb 13;62(2):98-104.

